# The First Chloroplast Genome Sequence of *Boswellia sacra*, a Resin-Producing Plant in Oman

**DOI:** 10.1371/journal.pone.0169794

**Published:** 2017-01-13

**Authors:** Abdul Latif Khan, Ahmed Al-Harrasi, Sajjad Asaf, Chang Eon Park, Gun-Seok Park, Abdur Rahim Khan, In-Jung Lee, Ahmed Al-Rawahi, Jae-Ho Shin

**Affiliations:** 1 UoN Chair of Oman’s Medicinal Plants & Marine Natural Products, University of Nizwa, Nizwa, Oman; 2 School of Applied Biosciences, Kyungpook National University, Daegu, Republic of Korea; Agriculture and Agri-Food Canada, CANADA

## Abstract

*Boswellia sacra* (Burseraceae), a keystone endemic species, is famous for the production of fragrant oleo-gum resin. However, the genetic make-up especially the genomic information about chloroplast is still unknown. Here, we described for the first time the chloroplast (cp) genome of *B*. *sacra*. The complete cp sequence revealed a circular genome of 160,543 bp size with 37.61% GC content. The cp genome is a typical quadripartite chloroplast structure with inverted repeats (IRs 26,763 bp) separated by small single copy (SSC; 18,962 bp) and large single copy (LSC; 88,055 bp) regions. *De novo* assembly and annotation showed the presence of 114 unique genes with 83 protein-coding regions. The phylogenetic analysis revealed that the *B*. *sacra* cp genome is closely related to the cp genome of *Azadirachta indica* and *Citrus sinensis*, while most of the syntenic differences were found in the non-coding regions. The pairwise distance among 76 shared genes of *B*. *sacra* and *A*. *indica* was highest for *atpA*, *rpl2*, *rps12* and *ycf1*. The cp genome of *B*. *sacra* reveals a novel genome, which could be used for further studied to understand its diversity, taxonomy and phylogeny.

## Introduction

A major distinguishing organelle of plant cells is the chloroplast, which was suggested to have originated from cyanobacteria through endosymbiosis [1–3]. The main role and function of the chloroplast is to perform photosynthesis and generate the building blocks required for plant growth and development [4,5]. Chloroplasts also participate in the biosynthesis of starch, fatty acids, pigments and amino acids [6]. Until recently, approximately 490 complete chloroplast (cp) genomes have been sequenced, and this information is publicly available (http://www.ncbi.nlm.nih.gov/ genome). Most of these assembled genomes are associated with economically important crop plants [7]. Chloroplast genome analysis and engineering, either alone or in combination with traditional breeding techniques, might provide information for the future development of novel plant sources to counteract various environmental stress tolerance issues and improve the level of human-derived benefits from the target plant [8]. The techniques might also provide information to improve the current understanding of major biosynthesis pathways and functions. The chloroplast (cp) genome contains important information in plant systematics and is maternally inherited in most angiosperms [9]. Thus, understanding these highly conserved structures could also reveal vital features for designing genetic markers.

Chloroplasts contain circular DNA with nearly 130 genes ranging in size from 72–217 kb [10–11]. Chloroplast DNA (cpDNA) is remarkably conserved in gene content and structure, envisaging important information for genome-wide studies of plant evolution [12]. Recent studies, using advanced genome sequencing techniques, have improved the prospects for resolving phylogenetic homogeneity at taxonomic levels and have enhanced the current understanding of the structural and functional evolution of economically important plants and their traits [12–15]. In the last decade, numerous studies have reported the results of chloroplast genome sequencing for economically and ecologically important tree species, such as *Eucommia ulmoides* [16], *Poplus cathayana* [17], *Quercus spinosa* [18], *Acacia ligulata* [19], *Pinus armandii* [20], *Cocos nucifera* [21], *Citrus aurantiifolia* [22], *Musa acuminata* [23], Norway spruce [24], etc. The elucidation of the chloroplast genomes of important tree species has facilitated to understand the evaluation of gene structure and has targeted conservation and propagation strategies [12–13].

**Fig 1:**
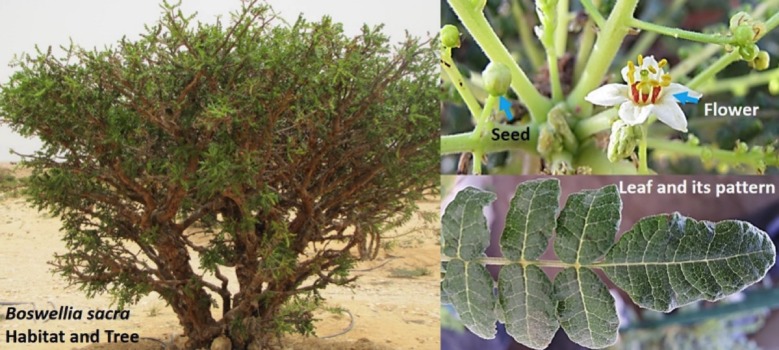
*Boswellia sacra*–habitat and leaf morphology. This tree grows wildly in the Dhofar region of Oman.

To understand the genome structure of chloroplasts, we have investigated the economically and culturally important frankincense-producing tree (*Boswellia sacra*; Burseraceae) [25]. There are approximately twenty *Boswellia* species, and *B*. *sacra* is an endemic species growing only in Dhofar region of Oman [25–26]. The trees grow in desert-woodlands (Fig 1) with meager amounts of water and nutrient availability [27], whereas the domesticated trees are supplied with water [28]. In response to the incisions by the local people, the tree activates its defense mechanism by producing resin [28]. The crystalline resin is used to create a fragrant smoke in homes and also sold in the market for income [26–27]. In addition to this, the tree has received much attention because of its medicinal uses in the Arabian region [29]. The essential oil and the boswellic acid or its derivatives possesses potent anticancer activities [30–32]. There is very scarce scientific knowledge exists regarding the genetic make-up and physiological functions of this endemic tree. Previously, Coppi et al. [31] has worked on the genetic diversity of *B*. *sacra* using ITS and ISSR analysis, where detailed genomic and conservation studies were suggested. Therefore, it is high time to further understand this keystone species. Hence, in the current study it was aimed to elucidate the complete chloroplast genome of *B*. *sacra* and determine the phylogenetic relationship of this tree with related species.

## Materials and Methods

### Ethics statement

The study site was managed by the Museum of the Frankincense Land (Salalah). The samples were collected from the Natural Park of Frankincense Tree, which is part of the Land of Frankincense sites, inscribed in the UNESCO’s World Cultural and Natural Heritage List. A permission was obtained from the Museum of the Frankincense Land to use the leaf part of the tree resources for research purposes. The trees used for sampling were treated ethically, and our study did not harm the local environment.

### Site description and plant growth conditions

Wadi Dawkah, Dhofar-Oman (17°25ʹ21ʹʹN; 54°00ʹ32ʹʹE) is a completely arid desert region with small sandstone hills. The annual mean temperature of the sampling area is ~35°C, and the annual rainfall is approximately 40 mm [22]. The temperature in summer reaches to ~48°C. Leaf samples were collected from healthy trees with the least number of incisions/wounds, immediately frozen in liquid nitrogen during the field trip to Wadi Dawkah and subsequently stored at -80°C until further analysis. The plant samples were collected by the first author in July 2015, which were identified by Plant Taxonomist Mohammed Al-Broumi, University of Nizwa. The specimens were deposited at University of Nizwa Herbarium Center with a voucher number (UC29).

### Chloroplast DNA extraction, sequencing and assembly

The fresh leaves from *Boswellia sacra* were washed with sterile distilled water to remove sand particles and soil debris. The chloroplast DNA was extracted by using a modified protocol of Shi et al. [33]. To prepare the sequencing library, 300 ng of extracted DNA was sheared to 400-bp by using the BioRuptor UCD-200 TS Sonication System (Diagenode Co., Belgium and Denville, NJ, USA). The fragmented DNA was used for subsequent library preparation using the Ion Xpress Fragment Library Kit (Life Technologies, Carlsbad, CA, USA). The DNA library was diluted to 4 pM, and emulsion PCR was performed using the Ion OneTouch System 2 (Life Technologies, Gaithersburg, MD, USA). The final sequencing library was loaded onto an Ion 316 v2 chip and sequenced on the Ion Torrent PGM for 850 flows using the Ion PGM Sequencing 400 Kit (Life Technologies, Gaithersburg, MD). The resultant sequence was *de novo* assembled and mapped with the reference genome [34]. The gaps identified were filled through another round of cp DNA extraction and sequencing. Following aforementioned procedures of cp DNA extraction, a second library was also prepared and sequenced separately to increase the raw data. The filtered sequence reads were assembled and aligned to the known cp genome sequences of Sapindales. At first, the filtered reads were *de novo* assembled into contigs using Mimicking Intelligent Read Assembly (MIRA 4.0) [35]. Second, the assembled contigs were mapped against the reference *Azadirachta indica* (accession number: NC 023792) through Map Reads to Reference Tool in CLC Genomics Workbench (version 8.5). The order of contigs was resolute according to the reference genomes. The gaps (7 to 150 bp) between the *de novo* contigs were replaced with consensus sequences of raw reads, which were later, re-mapped to the reference genomes. Any additional gaps were either filled manually by mapping tools in CLC Genomics Workbench (version 8.5) or using GapCloser (http://soap.genomics.org.cn/index.html).

### Chloroplast gene annotation and sequence analyses

Genome annotation was performed by using Dual Organellar GenoMe Annotator (DOGMA) [36], tRNAscan-SE v.1.21 [37] and Basic Local Alignment Search Tool (BLAST) [38] from the National Center for Biotechnology Information (NCBI). The using MAUVE v2.4.0 software [39] was used to compare the genes and sequences of the *de novo* assembled *B*. *sacra* cp genome sequence and *Azadirachta indica* cp genome sequence, as a reference. In addition, full alignments with annotations were visualized using the VISTA viewer [40].

### Repeat sequence analysis

MISA (http://pgrc.ipk-gatersleben.de/misa/) was used to analyze the types and positions of various simple sequence repeats (SSRs) in *B*. *sacra*. The results of this analysis were also correlated with the reference cp genome of *A*. *indica* according to the methods of Su et al. [22], where a minimum number of repeats were set at 10, 5, 4, 3, 3, and 3 for di-, tri-, tetra-, penta-, and hexanucleotides. For long repeats, the REPuter program [41] was used to identify the number and location of direct and inverted repeats. Minimums repeat size of 30 bp and sequence identity greater than 90% were used. Tandem repeats in the *B*. *sacra* cp genome were identified using Tandem Repeats Finder version 4.07 b [42] with the default settings.

Complete cp genomes as well as a separate partition using only 76 shared genes were employed to analyze the average pairwise sequence divergence for *B*. *sacra* and *Azadirachta indica*. Missing and ambiguous gene annotations were confirmed by comparative sequence analysis after a multiple sequence alignment and gene order comparison [43]. These regions were aligned using MAFFT (version 7.222) [44] with the default parameters. Kimura’s two-parameter (K2P) model was selected to calculate pairwise sequence divergences [45]. The *indel* (insertion and deletion) polymorphism of shared genes were calculated using DnaSP 5.10.01 [46]. A custom Python script (https://www.biostars.org/p/119214/) based on single-nucleotide polymorphism (SNP) definition (a variation in a single nucleotide that occurs at a specific position in the genome) was employed to call SNPs. In addition, the SNPs in coding regions were classified in two ways: synonymous and nonsynonymous as shown by Guo et al. [47]. The number of synonymous (S) and nonsynonymous (N) substitutions for the *B*. *sacra* and *Azadirachta indica* was performed on MEGA 7.0 [48].

### Phylogenetic analysis

Phylogenetic analysis of the *Boswellia sacra* cp genome was performed using Create Alignment and Create Tree tools in CLC genomics workbench version 8.5 (Gap open cost = 10.0, Gap extension cost = 1.0, Tree construction method = Neighbor Joining, Nucleotide distance measure = Jukes-Cantor) [49]. A total of 32 Malvidae species were selected as in-groups, whereas *Vitis venifera* was used as an outgroup. The accession numbers are provided in S1 Table. Complete cp genomes were used, and the bootstrap supports were estimated from 100 re-sampled alignments. Four methods were employed to construct phylogenetic trees from 32 cp genomes, including Bayesian inference (BI) implemented with MrBayes 3.12 [50], maximum parsimony (MP) with PAUP 4.0 [51], and maximum likelihood (ML), maximum parsimony (MP), posterior probabilities (PP) and neighbor-joining (NJ) with MEGA 7 [48].

## Results and Discussion

### General features of the *Boswellia sacra* chloroplast genome

Genome sequencing and assembly of Ion Torrent PGM sequencing produced 286.8 Mb of data for chloroplast genome of *Boswellia sacra*. A total of 373,248 reads (or 112,936,195 bp) were *de novo* assembled showing at total of 703x cp genome coverage. The chloroplast genome sequence quality was re-confirmed through repeating library preparation and sequencing of the extracted cpDNA from *B*. *sacra* tree. This result was sufficient to produce a single consensus sequence representing the complete chloroplast genome of *Boswellia sacra*. The *de novo* assembled genome was referenced with already reported cp genomes of *Sapindales* such as *Citrus sinensis* and *Azadirachta indica*. The gaps between the *de novo* contigs were replaced with consensus sequences of the raw reads and re-mapped to the reference genome. The sequences of the chloroplast genomes were deposited in GenBank (accession numbers: KT934315).

**Fig 2:**
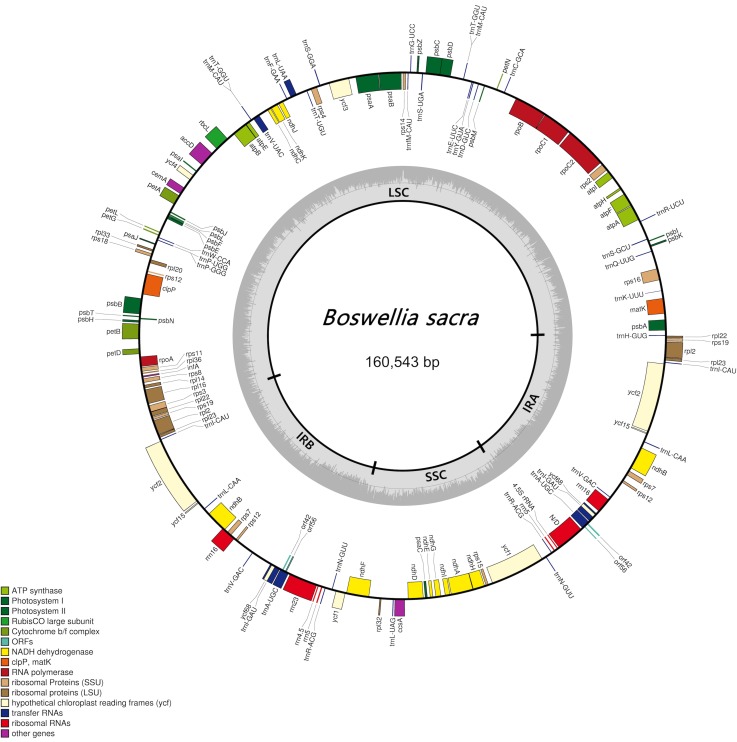
Gene map of the *Boswellia sacra* chloroplast genome. A pair of thick lines in the inside circle represents the inverted repeats (IRa and IRb; 26,763 bp each), separating the large single copy region (LSC; 88,055 bp) from the small single copy region (SSC; 18962 bp). The genes drawn inside the circle are transcribed clockwise, while those drawn outside the circle are transcribed counterclockwise.

The complete chloroplast genome was 160,543 bp in size, comprising a large single copy (LSC) region of 88,055 bp and a small single copy (SSC) region of 18,962 bp. In addition, a pair of inverted repeats (IRa and IRb) of 26,763 bp each (Fig 2) was also revealed. The annotation analysis revealed 114 different genes, with 83 protein-coding genes. The number of tRNA and rRNA genes was 27 and 4, respectively (Table 1). Among these, 24 genes were duplicated in the IR regions. The cp genome size of *B*. *sacra* was almost similar to *Azadirachta indica* (160,737 bp) and *Citrus sinensis* (160,129 bp). However, the size was almost 380 bp higher than closer relative in Order *Sapindales* i.e. *Citrus aurantifolia* (159,893 bp). In case of other arid-land trees, *B*. *sacra* size was higher than *Acacia ligulata* (158,724 bp) as shown in the previous studies of cp genomes of these plants [19, 22, 52]. This structure is consistent with the results of previous restriction mapping study [53], although the total lengths slightly differed.

**Table 1:** Summary of the chloroplast genome characteristics in *Boswellia sacra* and *Azadirachta indica*.

Attribute	*Boswellia sacra* (KT934315)	*Azadirachta indica* (NC023792)
Size (bp)	160,543	160,737
Overall GC content (%)	37.6	37.5
LSC size in bp (% total)	88,055 (54.8%)	88,136 (54.8%)
SSC size in bp (% total)	18,962 (11.8%)	18,635 (11.6%)
IR size in bp (% total)^a^	26,763 (16.7%)	26,983 (16.8%)
Protein-coding regions size in bp (% total)	80,289 (50.0%)	79,685 (49.6%)
rRNA and tRNA size in bp (% total)	11,978 (7.5%)	11,863 (7.38%)
Introns size in bp (% total)	17,621 (11.0%)	18,993 (11.8%)
Intergenic spacer size in bp (% total)	57,025 (35.5%)	52,699 (32.8%)
Number of different genes	114	112^b^
Number of different protein-coding genes	83	78
Number of different rRNA genes	4	4
Number of different tRNA genes	27	30
Number of different genes duplicated by IR	24	19
Number of different genes with introns	16	17

^a^Each cp genome contains two copies of inverted repeats (IRs).

^b^According to the original annotation, infA, ycf15, ycf68, orf42, orf56 not including.

Most of the essential photosynthesis-related genes were identified in the cp genome (Table 2; S2 Table). Twenty genes related to photosystem I and II, seventeen genes related to ATP synthase NADH dehydrogenase, six genes associated with the cytochrome b/f complex, a *large subunit rbcL*, two ORF, and other important genes related to ATP-dependent protease/ATP binding subunit (*clpP*), translational initiation factor (*infA*), Maturase K (*matK*), envelope membrane protein (*cemA*), subunit of acetyl-Co-Acarboxylase (*accD*), and c-type cytochrome synthesis gene (*ccsA*) were identified during annotation. The existence of chloroplast-encoded *clpP* in *B*. *sacra* suggested that this gene is indispensable for cell survival. Though this needs further verifications, but the presence of *clpP* indicates a better system to combat severe heat and drought stress in *B*. *sacra*, whereas *Cicer arietinum*, *Lathyrus sativus*, *and Pisum sativum* have lost an intron in both *clpP1* and *rps12* [54]. The *clpP* gene has also been identified in *Acacia ligulata* [19], *Trifolium subterraneum* [55], *Camellia* sp. [56], Actinidiaceae [57] and *Wollemia nobilis* [58].

**Table 2:** List of Genes found in cp genome of *Boswellia sacra*.

Group of gene	Name of gene
**Photosystem I**	*psaA*,*psaB*,*psaC*,*psaI*,*psaJ*
**Photosystem II**	*psbA*,*psbB*,*psbC*,*psbD*,*psbE*,*psbF*,*psbH*,*psbI*,*psbJ*,*psbK*,*psbL*,*psbM*,*psbN*,*psbT*, *psbZ*
**Cytochrome b/f complex**	*petA*,*petB*,*petD*,*petG*,*petL*,*petN*
**ATP synthase**	*atpA*,*atpB*,*atpE*,*atpF*,*atpH*,*atpI*
**NADH dehydrogenase**	*ndhA*,*ndhB*,*ndhC*,*ndhD*,*ndhE*,*ndhF*,*ndhG*,*ndhH*,*ndhI*,*ndhJ*,*ndhK*
**RubisCO**	*large subunit rbcL*
**RNA polymerase**	*rpoA*,*rpoB*,*rpoC1*,*rpoC2*
**Ribosomal proteins (SSU)**	*rps2*,*rps3*,*rps4*,*rps7*,*rps8*,*rps11*,*rps12*,*rps12_3end*,*rps14*,*rps15*,*rps16*,*rps18*,*rps19*
**Ribosomal proteins (LSU)**	*rpl2*,*rpl14*,*rpl16*,*rpl20*,*rpl22*,*rpl23*,*rpl32*,*rpl33*,*rpl36*
**Other genes**	*clpP*,*matK*,*accD*,*ccsA*,*infA*,*cemA*
**hypothetical chloroplast reading frames**	*ycf1*,*ycf2*,*ycf3*,*ycf4*,*ycf15*,*ycf68*
**ORFs**	*orf42*,*orf56*
**Transfer RNAs**	*trnA-UGC*,*trnC-GCA*,*trnD-GUC*,*trnE-UUC*,*trnF-GAA*,*trnG-UCC*,*trnH-GUG*,*trnI-CAU*,*trnI-GAU*, *trnK-UUU*,*trnL-CAA*,*trnL-UAG*,*trnM-CAU*,*trnN-GUU*, *trnP-GGG*,*trnP-UGG*,*trnQ-UUG*, *trnR-ACG*,*trnR-UCU*,*trnS-GCU*,*trnS-GGA*,*trnT-GGU*,*trnV-GAC*,*trnV-UAC*,*trnW-CCA*,*trnY-GUA*
**Ribosomal RNAs**	*rrn4*.*5*,*rrn5*,*rrn16*,*rrn23*

### General comparisons of *Boswellia sacra* and *Azadirachta indica*

The general characteristics of the *B*. *sacra* and *A*. *indica* cp genomes are presented in Table 1. The results showed a high level of similarity in the overall composition of the two cp genomes. The *B*. *sacra* cp genome had approximately 37.61% GC content, which is higher than that of *A*. *indica* (37.5%). In the *B*. *sacra* cp genome, the intergenic regions, introns, and genic regions account for ca. 33.5%, 9.6%, and 57.5%, respectively, as shown in Tables 1 & 2. The pairwise alignment of these two cp genomes revealed approximately 9.52% sequence divergence (Table 3; S3 Table; S4 Table; S5 Table) comprising 8,929 substitutions (5.56%) and 3,971 *Indels* (3.96%). The sizes of the LSC and SSC regions were somehow different between these two cp genomes, primarily reflecting the large index in each region. The 10 most divergent intergenic regions included the spacer between *trnH—GUG-psbA* (457 bp, 40.54% divergence), *accD—psaI* (746 bp, 37.27% divergence), *atpH—atpI* (1,167 bp, 33.94% divergence), *psbZ—trnG-UCC* (546 bp, 28.14% divergence), *trnK—UUU-rps16* (1,034 bp, 26.40% divergence), *petN—psbM* (1,139 bp, 36.21% divergence), *ycf4—cemA* (942 bp, 26.69% divergence), *ccsA—ndhD* (307 bp, 25.24% divergence), *trnR-UCU—atpA* (198 bp, 28.97% divergence) and *trnL-UAG—ccsA* (111 bp, 33.05% divergence). These findings suggest that a deletion might have occurred in this region in *A*. *indica* after the divergence of *A*. *indica* and *B*. *sacra*.

**Table 3:** Differences between the *B*. *sacra* and *A*. *indica* cp genomes.

**Indel**				
		Length (bp)	Count	
		1	2,743	
		2–10	1,216	
		11–21	12	
	Sum	6,353	3,971	Percentage^a^: 3.96%
**Substitution**				
		Type	Count	
		A<->T	1,571	
		C<->G	837	
		A<->C	1,416	
		T<->C	1,828	
		A<->G	1,777	
		T<->G	1,500	
	Sum		8,929	Percentage^a^: 5.56%
**10 most divergent intergenic regions**				
		Region	length^b^ (bp)	Pairwise distance
		trnH—GUG-psbA	457	0.47
		accD—psaI	746	0.32
		atpH—atpI	1,167	0.31
		psbZ—trnG-UCC	546	0.30
		trnK—UUU-rps16	1,034	0.25
		petN—psbM	1,139	0.25
		ycf4—cemA	942	0.25
		ccsA—ndhD	307	0.24
		trnR-UCU—atpA	198	0.23
		trnL-UAG—ccsA	111	0.21

^a^Relative to the length of *B*. *sacra*.

^b^Length in *B*. *sacra*.

In case of *indels*, the cp genome of *B*. *sacra* was highly conserved, however, in total 25 loci were identified with most variable regions. *ycf1* was highly variable region showing a 4354 *indel* sites, followed by *atpA* with 124 *indel* sites. The average *indel* length for *ycf1* was 174 bp, followed by *rpl14* with deletion of 25.5 bp. On the other hand, the *indel* diversity per site was highest for *rpl2* (S6 Table). We also compared the cp genomes of *B*. *sacra* and *A*. *indica* and calculated the average pairwise sequence divergence among 76 shared genes (S7 Table). Of these, the *B*. *sacra* genome has 0.066 average sequence divergence. Furthermore, among the genes identified to show highest level of divergence were *atpA*, *rpl2*, *rps12*, *ycf1*, *infA*, *rpl22*, *rpl32*, *rpl20*, *ndhF* and *rps8* (S3 Fig). The highest average sequence distance was found for *atpA* (0.852), followed by *rps11* (0.301).

The *trnH/psbA* intergenic region has been studied as a barcode of differentiation at the species level in the plastids of inland plants, such as *Araucaria* [57–59]. This region, highly variable in length and sequence, is an on-coding region flanked by two conserved coding regions, i.e., *psbA*, which encodes photosystem II protein D1, and *trnH-GUG*, which functions in histidine translation [12–13]. The results revealed a 457 bp sequence *trnH*/*psbA* intergenic region in *B*. *sacra*. Yap et al. [57] suggested that this sequence was not present in *Agathis dammara* compared with *Wollemia nobili*. Previous detailed BLAST analyses have shown that this *Indel* is present in nineteen *Araucaria* species, including *W*. *nobili* [57]. In addition, psbA protects photosystem II from oxidative stress developed through drought. A previous report of Huo et al. [60] demonstrated that the overexpression of the *psbA* gene improves drought tolerance in tobacco plant. The occurrence of *psbA* in *B*. *sacra* suggests a strong ability to counteract oxidative stress, as this tree frequently encounters low-water conditions, however, this still needs further in-depth investigations.

### Synteny between *B*. *sacra* and *A*. *indica*

**Fig 3:**
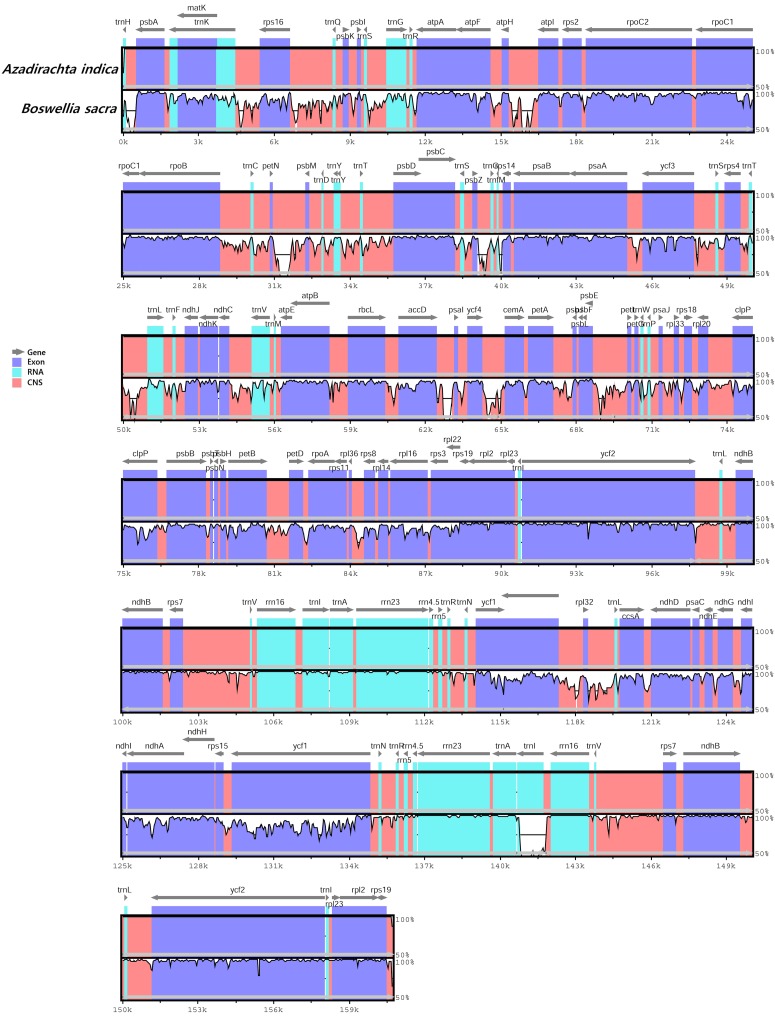
Visualization alignment of chloroplast genome sequences of *B*. *sacra* and *A*. *indica*. VISTA based similarity graphical information portraying sequence identity of *B*. *sacra* with reference *A*. *indica* cp genomes. Thick black lines show the inverted repeats (IRs) in the chloroplast genomes. Genome regions are color-coded as protein coding, rRNA coding, tRNA coding or conserved noncoding sequences (CNS) whereas arrows show the gene presence.

A syntenic structure was also visualized in the MAUVE alignment for both cp genomes as shown in S1 Fig. The MAUVE alignment showed that there are two locally collinear blocks (LCBs) in the chloroplast genomes of *B*. *sacra* and *A*. *indica*. These regions showed homology in genome organization between the two species, although the sequences are inverted relative to each other. The LCBs of protein coding, tRNA, and rRNA genes showed a similar synteny for both cp genomes. Similarly, the two cp genomes were run global alignment through VISTA to understand the comparative genomic organization [40]. The results of the alignment showed that both are having high sequence similarity. However, the marked differences were observed between the non-coding regions (*petN* and *psbM*; *accD* and *psal*; *ycf4* and *cemA*; *rpl32* and *trnL*; *atpH* and *atpl*). The most divergent in coding regions were *trnl*, *matK*, *clpP*, *rpl33*, *accD*, *rps3*, *ndh (A*, *H*, *D and G)*, *ycf3* and *ycf1* (Fig 3). Leliaert and Lopez-Bautista [61] revealed a similar homogenous matrix in cp genome sequencing between *Bryopsis plumosa* and *Tydemania expeditions*. However, dissimilarity in the synteny of some fragments of both cp genomes was observed, and this high dissimilarity is not unexpected, as high erraticism in the architecture of cpDNA has been observed among various species of congeneric taxa, such as picoplanktonic species, and *Trebouxiophyceae* and *Chlorophyceae* [62–63].

### Analysis of repetitive sequences

Repetitive sequencing in cp genomes plays an important function and enhances the current understanding of the evolutionary aspects of plant species [62–63]. Mononucleotide microsatellite length polymorphisms have been used as markers to understand the evolutionary history of cp genomes, reflecting the high variability rates of these anomalies [56]. In the present study, a total of 109 simple sequence repeat (SSR) loci were observed in the *B*. *sacra* chloroplast genome, sharing 1,256 bp (ca. 0.78%) of the total sequence (Table 4). Most of the SSRs were identified in intergenic regions, and some SSRs were identified in coding areas, such as *rpoC2* and *ycf1*, which could also be used as molecular markers for population diversity studies [63]. A total 39 large repeats of more than 30 bp in length were identified in the *B*. *sacra* cp genome (Table 5).

**Table 4:** List of simple sequence repeats.

Repeat unit	Length (bp)	Number of SSRs	Start position^a^
**AT**	10	5	**21733** (*rpoC2*); 50519; 63260; 120682; **122312** (*ndhD*)
	12	2	72021; 118991
**TA**	10	1	**50086**
	12	1	49380
**TC**	10	1	**65118** (*cemA*)
**AAG**	12	1	**98114**
**ATA**	12	3	**52097**; 57925 (*atpB*); 70908
**CTT**	12	1	**150474**
**TAT**	12	1	50027
**TTA**	12	2	**54843**; 131240 (*ycf1*)
**AAAT**	12	1	6383
**AATT**	12	1	117168
**ATAG**	12	1	34089
**CATT**	12	1	**131003** (*ycf1*)
**CTTT**	12	1	51864
**TATC**	12	1	38353
**TTTC**	12	1	125090
**ATATGA**	18	1	118904

^a^The SSR containing coding regions are indicated in parentheses. SSRs that are identical in the *A*. *indica* chloroplast genome are highlighted in bold.

**Table 5:** List of long repeat sequence.

**Repeat Size**	**Type**^a^	**Start position of 1st repeat**	**Start position the repeat found in other region**	**Location**^b^	**Region**
**30**	P	9578	48299	***trnS-GCU*, *trnS-GGA***	LSC
**30**	D	30965	31304	IGS (*petN*—*psbM*)	LSC
**30**	D, P	95755	95773, 152797, 152815	***ycf2***	IR
**31**	D	50042	50183	IGS (*trnT-UGU*—*trnL-UAA*)	LSC
**32**	D	15474	15696, 16029	IGS (*atpH—atpI*)	LSC
**34**	P	5863	5863	intron (*rps16*)	LSC
**36**	D, P	103143	125725, 145421	**IGS (*rps12—trnV-GAC*), intron (*ndhA*), IGS (*trnV-GAC—rps12*)**	IR, SSC
**40**	D	64428	64797	IGS (*ycf4—cemA*)	LSC
**42**	D	49991	50135	IGS (*trnT-UGU—trnL-UAA*)	LSC
**42**	D	64386	64759	IGS (*ycf4—cemA*)	LSC
**52**	P	444	444	IGS (*trnH-GUG—psbA*)	LSC
**56**	P	31815	31815	**IGS (*petN—psbM*)**	LSC
**65**	D	15507	^16061^^*^	IGS (*atpH—atpI*)	LSC
**69**	D	31107	31522	IGS (*petN—psbM*)	LSC
**76**	D	41575	43799	***psaB*, *psaA***	LSC
**109**	D	64274	64648	IGS (*ycf4—cemA*)	LSC
**155**	D	15417	15639	IGS (*atpH—atpI*)	LSC
**204**	D	15729	^16061^^*^	IGS (*atpH—atpI*)	LSC
**251**	D	62442	62769	*IGS (accD—psaI)*, *psaI*	LSC

^a^D: direct repeat; P: palindrome inverted repeat.

^b^IGS: intergenic spacer region. Sequences conserved in the *A*. *indica* chloroplast genome are highlighted in bold.

*The 65 bp sequence is truncated copy of 204 bp sequence.

In case of tandem repeat, some of the conifers have been noted for the presence of large number of such repeats [64]. These repeats have been associated with gene duplication and chloroplast rearrangement [65]. The results of tandem repeat analysis showed a total 30 repeats (Table 6). The highest size of repeats was 76 bp in length *accD* in the coding region whereas the lowest was 12 bp *trnK/rps16* in the intergenic spacer region, with average copy number 2.4. The percent of *indels* were ranged from 0 to 18 (Table 6). A majority of these repeats were located in intergenic spacers, while five repeat sequences were identified in the coding regions of *trnS-GCU*, *trnS-GGA*, *ycf2*, *psaB* and *psaA*. Eight long repeats were identified in *A*. *indica*, suggesting that these repeats might be well distributed among Sapindales. In a recent study, Addisalem et al. [65] developed the first set of 46 SSR markers and estimated the genome size for *B*. *papyrifera* as 705 Mb, representing the first genome sequence analysis for any species of this genus. However, the current knowledge of the genomics and transcriptomics of this keystone species remains limited.

**Table 6:** Distribution of tandem repeats in the *B*. *sacra* chloroplast genome.

Indices	Repeat Length	Copy Number	Percent Indels	Percent Matches	Location
347—387	16	2.6	11	88	*trnH/psbA* (IGS)
4566—4594	12	2.4	0	100	*trnK/rps16* (IGS)
4819—4874	19	2.9	5	78	*trnK/rps16* (IGS)
7784—7814	16	1.9	0	100	*rps16/trnQ*(IGS)
8690—8726	18	2.1	0	94	*trnQ/psbK*(IGS)
9763—9828	27	2.4	5	84	*trnS/trnR* (IGS)
9783—9831	14	3.6	5	83	*trnS/trnR* (IGS)
9779—9867	27	3.5	13	85	*trnS/trnR* (IGS)
9863—9892	14	2.1	0	100	*trnS/trnR* (IGS)
11501—11572	31	2.5	18	81	*trnR/atpA*(IGS)
11504—11573	25	2.7	17	76	*trnR/atpA*(IGS)
11535—11583	22	2.4	12	80	*trnR/atpA*(IGS)
16355—16385	14	2.2	0	94	*atpH/atpI* (IGS)
29582—29606	12	2.1	0	100	*rpoB/trnC*(IGS)
31751—31776	13	2	0	100	*petN/psbM*(IGS)
49997—50046	18	2.9	11	82	*trnT/trnL*(IGS)
60254—60278	12	2.1	0	100	*rbcL/accD*(IGS)
62281—62429	76	2	5	88	*accD*(CDS)
67916—67954	19	2.1	4	90	*psbJ/psbL*(IGS)
69384—69454	12	6.1	18	72	*psbE/petL*(IGS)
72176—72214	21	1.9	5	94	*rps18* (CDS)
73287—73312	13	2	0	100	*rpl20/rps12*(IGS)
95746—95802	18	3.2	0	97	*ycf2*(CDS)
112196—112261	32	2.1	0	97	*rrn 4*.*5 /rrn5*(IGS)
113767—113809	20	2.2	0	100	*trnN/ycf1*(IGS)
119576—119605	15	2	0	93	*ccsA*(CDS)
129059—129085	13	2.1	0	100	*rps15/ycf1*(IGS)
134790—134832	20	2.2	0	100	*ycf1*(CDS)
136338—136403	32	2.1	0	97	*rrn5/rrn4*.*5*(IGS)
152797—152853	18	3.2	0	97	*ycf2*(CDS)

In case of single-nucleotide polymorphism (SNP) (a variation in a single nucleotide that occurs at a specific position in the genome), maximum likelihood analysis of natural selection codon-by-codon, was performed to estimate the number of inferred synonymous (s) and nonsynonymous (n) substitutions which are presented along with the numbers of sites that are estimated to be synonymous (S) and non-synonymous (N) [66]. These estimates are produced using the joint Maximum Likelihood reconstructions of ancestral states under a Muse-Gaut Model [67] of nucleotide substitution. A total to 8,301 positions were noted in the chloroplast genomes of *B*. *sacra*, *A*. *indica* and *Citrus sinensis*. In case of codons, TCT, GTT, GGA and CGT showed high n, S, N and *dN-dS* (S8 Table). However, the *P* value was less than 0.05 showing a significant substitution among cp genomes. The normalized *dN/dS* ratio was calculated for the three cp genomes which showed a positive selection ranging from 0~10.17. The number of segregating sites were 14,249 in three cp genomes with a nucleotide diversity was 0.063. The nucleotide frequencies are 30.59% (A), 31.21% (T/U), 19.49% (C), and 18.71% (G) in the cp genomes. The transition/transversion rate ratios are k1 = 2.149 (purines) and k2 = 2.072 (pyrimidines). The overall transition/transversion bias is R = 0.996. These shows that *B*. *sacra*, *A*. *indica* and *Citrus sinensis* species with long generation times, and both exhibit little changes in the structural organization of the chloroplast genomes. This could further reveal how these species evolved at low rates. Similar perspectives were shown for the chloroplast genomes of five *Quercus* species [66]. The estimation of synonymous and non-synonymous substitution rates may play an important role in understanding the dynamics of molecular evolution, and non-synonymous substitutions could be subject to natural selection during the evolutionary process [66–68].

### Evolution of *orf56* and *ycf68* within *sapindales*

**Fig 4:**
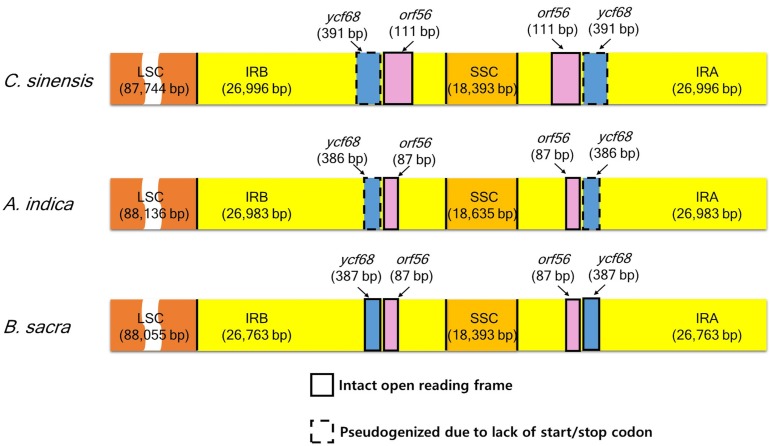
Comparison of the border positions of the LSC, SSC, and IR regions in three Sapindales species.

We compared the *orf56* and *ycf68* genes of three tree species viz. *B*. *sacra*, *A*. *indica* and *C*. *sinensis* (Fig 4), revealing the presence of these genes in the three species. The *orf56* gene is present in an intron in the *trnA-UGC* gene, which contains one homologous sequence, known as mitochondrial ACR-toxin sensitivity gene in *Citrus* trees [22, 69]. Interestingly, the full-length *orf56* gene sequence was different but was intact in all three species. Goremykin et al. [70] previously reported that the ACR-toxin sensitivity gene was transferred between plastid and mitochondrial genomes [22].

The *ycf68* gene, in the *trnI-GAU* intron of the IR region, showed high similarity between *C*. *sinensis* and *B*. *sacra*. However, an additional T-insertion near the C-terminus was abolished at the stop codon [22]. *A*. *indica* has a T-deletion near the C-terminus abolished at the stop codon in the same position. Comparatively, an intact *ycf68* gene was detected in *B*. *sacra* (Fig 4). Raubeson et al. [71] suggested that *ycf68* is not a protein-coding gene based on the lack of intron folding patterns. The high levels of sequence conservation among the ORF of identified homologs indicates true functionality [72]. However, the function of this putative gene still remains unclear and needs further investigation.

### Phylogenetic analysis of *B*. *sacra*

To obtain insight into the position of *B*. *sacra* within the Malvidae, we generated datasets of 32 completely sequenced chloroplast genomes, using *Vitis venefera* as an outgroup (S1 Table). We conducted a phylogenetic analysis of the *B*. *sacra* cp genome with the cp genomes from 8 species of Myrtaceae *(Corymbia eximia*, *Angophora costata*, *Eucalyptus umbra*, *E*. *deglupta*, *E*. *globulus*, *Stockwellia quadrifida*, *Syzygium cumini* and *Allosyncarpia ternata*), 2 species from Melianthaceae (*Francoa sonchifolia* and *Theorbroma cacao*), 3 species from Malvaceae (*Gossypium incanum*, *G*. *tomentosum*, and *G*. *hirsutum*), 15 species from Brassicaceae *(Barbarea verna*, *Nasturtium officinale*, *Lepidium virginicum*, *Pachycladon enysii*, *P*. *cheesemanii*, *Crucihimalaya wallichii*, *Olimarabidopsis pumila*, *Capsella bursa-pastoris*, *Arabidopsis thaliana*, *Lobularia maritima*, *Brassica napus*, *Arabis hirsuta*, *Draba nemorosa*, *Aethionema cordifolium* and *Aethionema grandiflorum*), one species from Caricaceae (*Carica papaya*) and one species from Rutaceae (*Citrus sinensis*).

**Fig 5:**
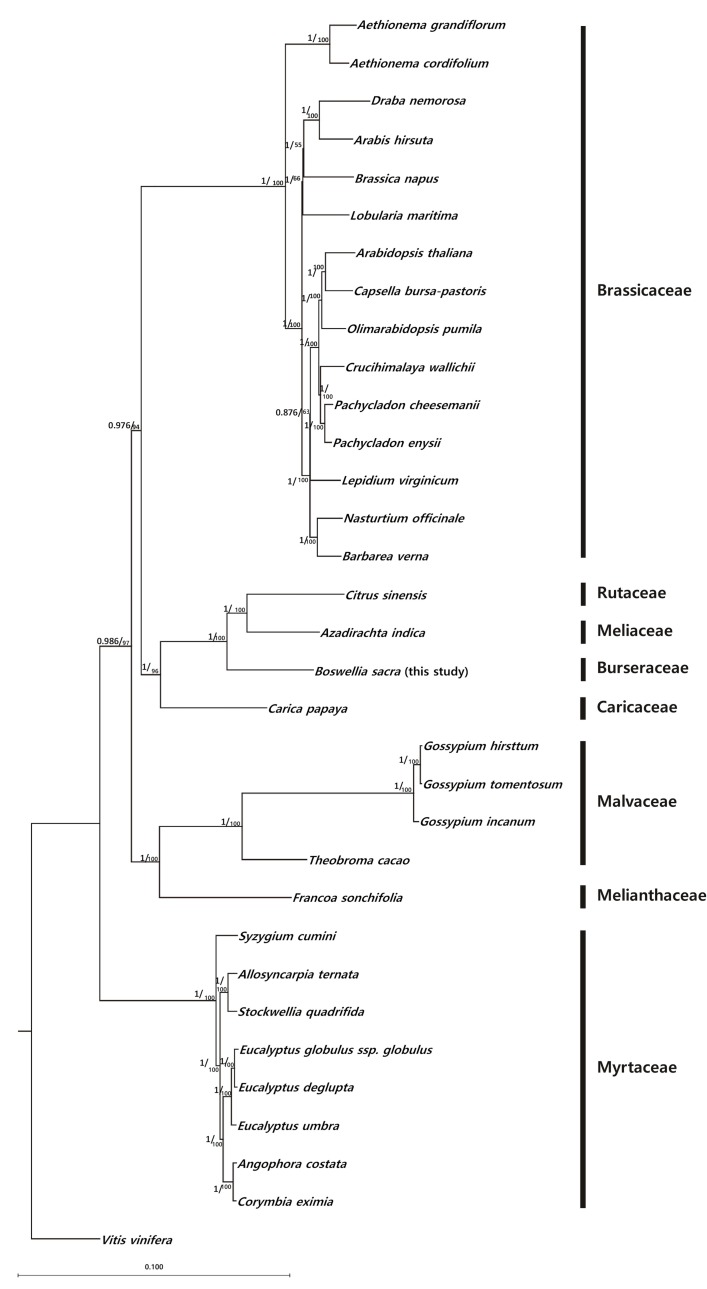
Neighbor-Joining phylogeny of the representative Malvidae lineages. The common grape vine (*Vitis vinifera*) is included as the outgroup to root the tree. A total of 33 complete chloroplast genomes were aligned. In total, 33 nodes were resolved. The position of *B*. *sacra* is shown in bold.

The phylogenetic analysis included some commercially important Sapindales plants and the more relevant *A*. *indica* species from Maliaceae. The neighbor-joining phylogenetic tree resolved 33 nodes, with strong bootstrap support of 55–100 (Fig 5). These results strongly support the position of the *B*. *sacra* within Sapindales. The phylogenetic trees obtained in the present study also indicate a close relationship between *B*. *sacra* and *A*. *indica* with high bootstrap support (100%). Krishnan et al. [73] suggested that both *A*. *indica* and *Citrus sinensis* form a homogenous group (100%), validating the conventional taxonomic classification of *A*. *indica*. Thus, the results of the phylogenetic analysis of the cp genome of both species showed the formation of a distinct cladogram with *B*. *sacra*, revealing new insights to understand the gene structure of these three plants.

The habitats of *B*. *sacra* and *A*. *indica* are different, however, the fact that both plants produce oleugum and belong to the same order suggests interesting prospects for broader evolutionary studies of Sapindales [74]. This phylogenetic relationship was further confirmed by constructing the maximum parsimony tree [48], suggesting a close homology with *C*. *sinensis* and *A*. *indica* (S2 Fig). The Bayesian Inference (BI) also highly supported as monophyletic (100/1.00) behavior *C*. *sinensis* and *A*. *indica*. Whereas a sister relationship of *B*. *sacra* was well supported (100/1.00). The posterior probabilities (PP) were also 1.0 for *B*. *sacra*, *C*. *sinensis* and *A*. *indica* (S4 Fig), suggesting a branching order as ((*C*. *sinensis*, *A*. *indica*), *B*. *sacra*). A similar phylogenomic reconstruction was also noticed by Wang et al. [16,75], where Actinidiaceae formed a monophyly relationship in Ericales.

## Conclusions

Using a combination of *de novo* assembly and reference genome analysis, we provided the first completely sequenced chloroplast genome of endemic *Boswellia sacra*. The genome organization and gene structure is almost typical to that of angiosperms plants. The main difference in the gene composition between the *B*. *sacra* and *A*. *indica* cp genomes was the protein-coding gene in the *trnH-GUG-psbA* region. Notably, eight shared SSR regions were identified in the *B*. *sacra* and *A*. *indica* cp genome comparison. Elucidating these regions could provide additional phylogenetic insights at the taxonomic level, and these results could be used to develop molecular markers [72]. Previously, Coppi et al. [31] suggested that different stands of tree populations growing in different areas showed significant variations in genetic distances. The findings of the present study are essential to envisage fresh in-depth intuitions into various aspects of *Boswellia* chloroplast genome evolution. The current new genomic datasets will enable further exploration of the genetic diversity of *Boswellia sacra* and the other 18 species of this genus.

## Supporting Information

S1 Table List of the complete chloroplast genome sequences included in the phylogenetic analysis. (XLSX)

S2 Table Functions and products of the chloroplast genes identified in *B*. *sacra*. (DOCX)

S3 Table Sequences of important gene fragments in *B*. *sacra* and comparison with *A*. *indica*. (XLSX)

S4 Table Simple Sequence Repeat analysis of *B*. *sacra*. (XLSX)

S5 Table Pairwise distances of the important regions identified in *B*. *sacra* and *A*. *indica*. (XLSX)

S6 Table Comparative assessment of *Indels B*. *sacra* and *A*. *indica*. (XLSX)

S7 Table Pairwise distance among the shared genes of *B*. *sacra* and *A*. *indica*. (XLSX)

S8 Table Comparisons of synonymous (S) and nonsynonymous (N), transitional substitutions are shown in bold and those of transversionsal substitutions in chloroplast genome of *B*. *sacra*, *A*. *indica and Citrus sinensis*. (XLS)

S1 Fig MAUVE alignment of the *Boswellia sacra* chloroplast genome. The *B*. *sacra* genome is shown on top as the reference genome. Within each of the alignments, local collinear blocks are represented as blocks of the same color connected with lines. Annotations are shown above and below the LCBs, protein-coding genes are indicated as white boxes, tRNA genes are shown in green, and rRNA genes are shown in red. The lowered position of a box indicates an inverted orientation.
(TIFF)

S2 Fig Maximum parsimony phylogeny of the representative cp genomes. The *Gossypium incanum* was included as an outgroup to root the tree. A total of 21 complete chloroplast genomes were aligned. In total, 21 nodes were resolved. The position of *B*. *sacra* is shown in green circular symbol.
(TIF)

S3 Fig Pairwise distances of shared 76 genes *B*. *sacra* and *A*. *indica*. (TIF)

S4 Fig Posterior probabilities out put of the selected cp genomes in relation with *B*. *sacra* and *A*. *indica*. (PDF)
