# Supplementary material for: The First Chloroplast Genome Sequence of Boswellia sacra, a Resin-Producing Plant in Oman
Source: PLoS One. 2017 Jan 13;12(1):e0169794. doi: 10.1371/journal.pone.0169794 (PMC5235384; doi:10.1371/journal.pone.0169794)
Supplement: S2 Table — (DOCX) [file pone.0169794.s002.docx]

**Table S2.** Chloroplast genes found in *B. sacra* and their function and product

| Gene (s) | Role | Product |
| --- | --- | --- |
| *acc* | fatty acid metabolism | c-type cytochrome synthesis protein; acetyl-CoA carboxylase biotin carboxyl carrier protein; acetyl-CoA carboxylase beta subunit; acyl carrier protein |
| *ycf* | Conserved Hypo | Hypothetical protein complex; ABC transporter; chloroplast, |
| *atp* | Energy metabolism | Adenosine triphosphate synthase |
| *ccs* | Biosyn of co-factors | Cytrochrome c biogenesis protein |
| *cem* | Transport membrane | chloroplast envelope membrane protein |
| *clp* | Cellular processes | ATP-dependent clp protease ATP binding subunit |
| *inf* | Hypothetical/uncharacterized | translation initiation factors |
| *lsc* | long single copy | Adenosine triphosphate synthase |
| *Mat* | Hypo/uncharac | maturase K |
| *ndh* | Energy metabolism | NADH- Plastoquinone reductase |
| *pet* | Energy metabolism | Cytochrome b6 complex protein family |
| *psa* | Photosynthesis | Photosystem I reaction center complex |
| *psb* | Photosynthesis | Photosystem II reaction complex |
| *RbcL* | Photosynthesis | ribulose bisphosphate carboxylase large chain |
| *rpl* | Translation | ribosomal protein involved in translation |
| *rpo* | Transcription | DNA-directed RNA polymerase alpha chain |
| *rps* | Translation | Ribosomal protein |
| *rrn* | rRNA | Plastid rRNA |
| *trn* | tRNA | Plastid tRNA |
