# Supplementary figures and images for: The First Chloroplast Genome Sequence of Boswellia sacra, a Resin-Producing Plant in Oman
Source: PLoS One. 2017 Jan 13;12(1):e0169794. doi: 10.1371/journal.pone.0169794 (PMC5235384; doi:10.1371/journal.pone.0169794)

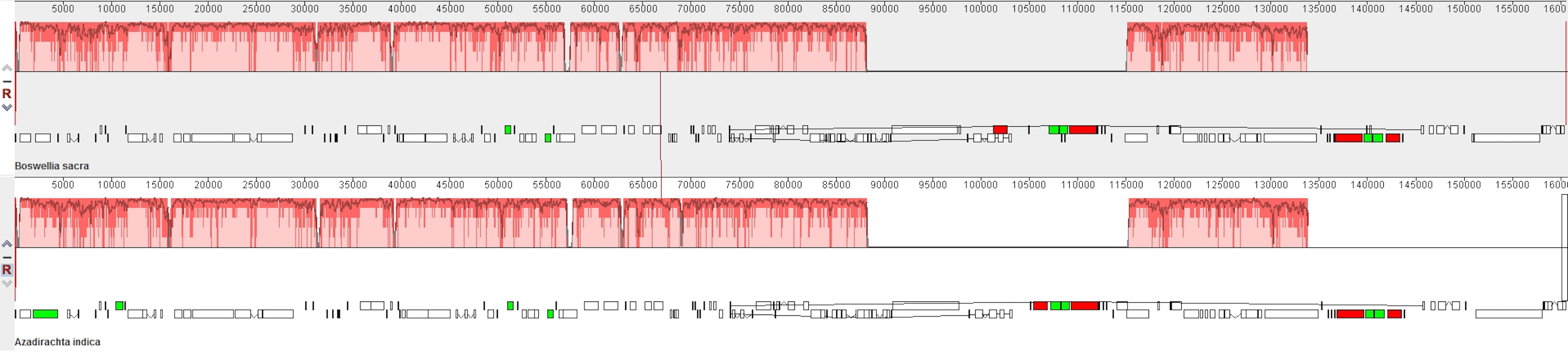

Supplement: S1 Fig — The B. sacra genome is shown on top as the reference genome. Within each of the alignments, local collinear blocks are represented as blocks of the same color connected with lines. Annotations are shown above and below the LCBs, protein-coding genes are indicated as white boxes, tRNA genes are shown in green, and rRNA genes are shown in red. The lowered position of a box indicates an inverted orientation. (TIFF) [file pone.0169794.s009.tiff]

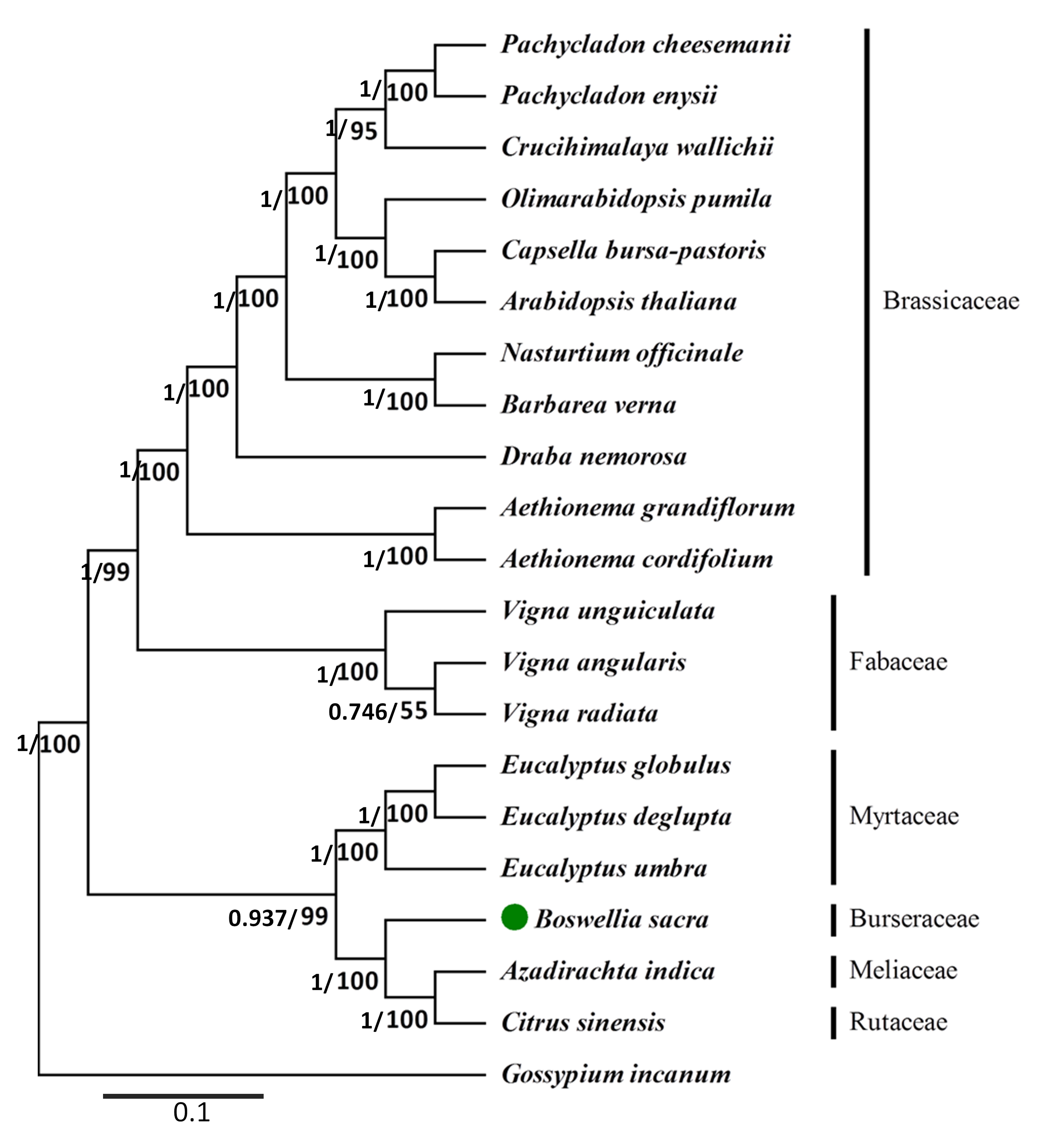

Supplement: S2 Fig — The Gossypium incanum was included as an outgroup to root the tree. A total of 21 complete chloroplast genomes were aligned. In total, 21 nodes were resolved. The position of B. sacra is shown in green circular symbol. (TIF) [file pone.0169794.s010.tif]

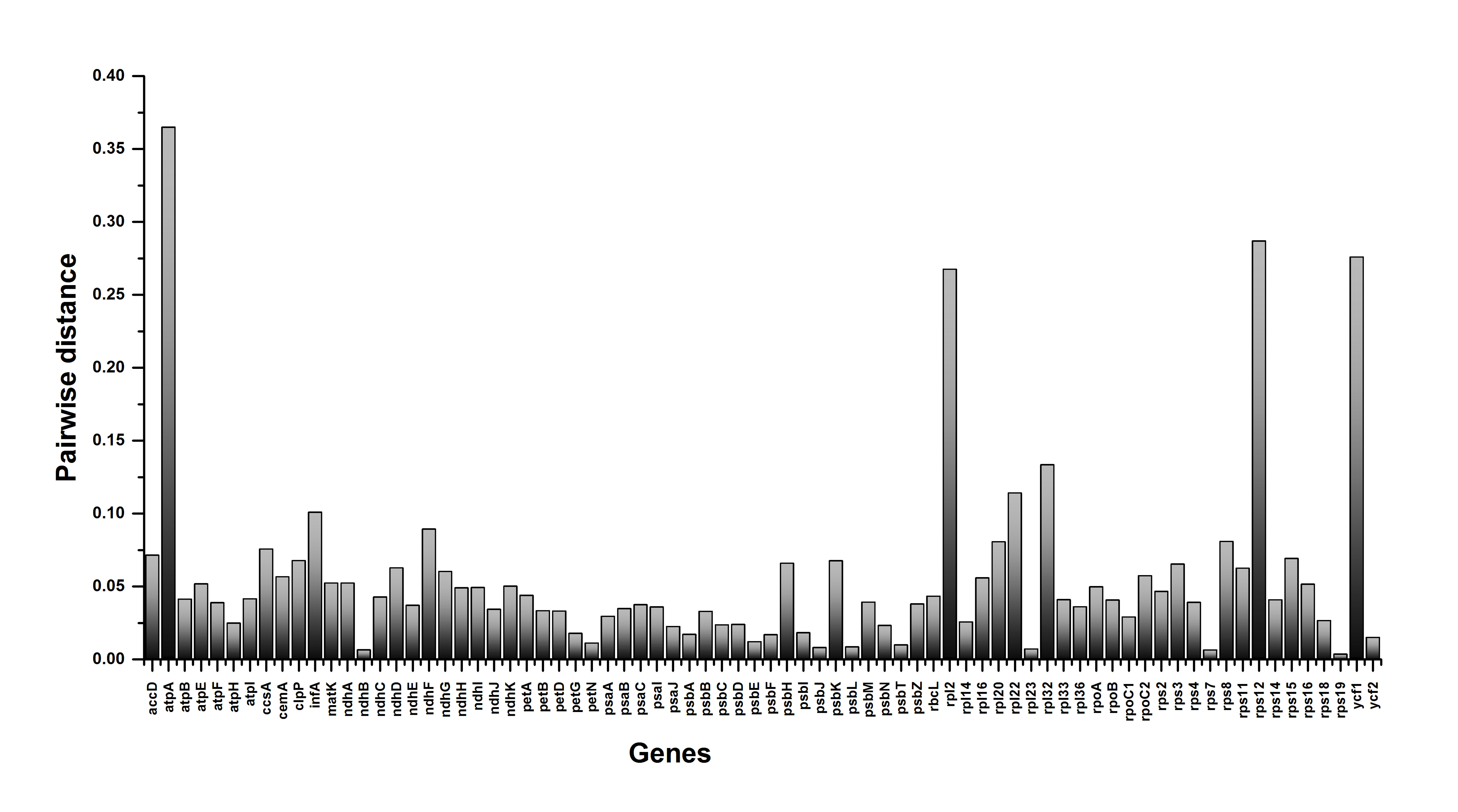

Supplement: S3 Fig — (TIF) [file pone.0169794.s011.tif]

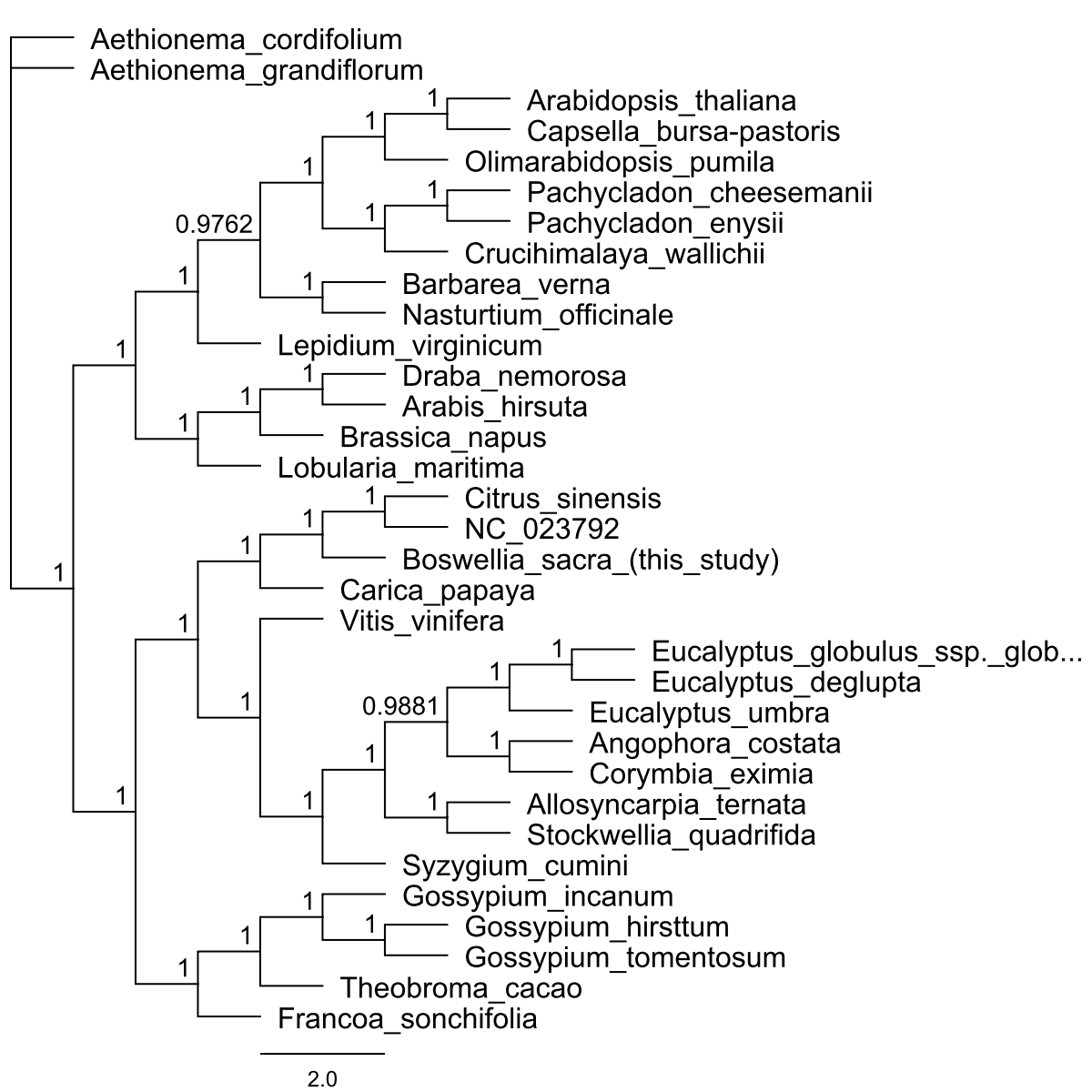

Supplement: S4 Fig — (PDF) [file pone.0169794.s012.pdf]
